# Heritability and social interaction in labor market outcomes: Evidence from the population of twins in the United States

**DOI:** 10.1073/pnas.2620927123

**Published:** 2026-09-08

**Authors:** David Card, Jesse Rothstein, Moises Yi

**Affiliations:** ^a^ Department of Economics, University of California, Berkeley, CA 94720-3880; ^b^ Center for Economic Studies, US Census Bureau, Suitland, MD 20746

**Keywords:** heritability, twins, intergenerational transmission

## Abstract

We use data on 44,000 twin pairs observed as adolescents in the 2000 Census, linked to their earnings 20 y later, to study the heritability of labor market outcomes. We extend the Classical Twins Design (CTD), which identifies heritability from contrasts between monozygotic and dizygotic twins, to settings where an analyst does not observe zygosity but can measure outcomes for same-sex and opposite-sex twins. We also allow for the presence of a family- and sex-specific component affecting same-sex siblings but only partly shared by opposite-sex siblings. Our extended model identifies heritability from the difference-in-differences of same-sex vs. opposite-sex twins vs. siblings. The estimates indicate strong heritability (h-sq = 0.36) of log earnings. Using an AKM [J. M. Abowd *et al.*, *Econometrica* **67**, 251–333 (1999)] decomposition that separates the person-specific component of earnings from employer-specific pay premiums, we find that both components are heritable. The heritability of employer pay premiums, however, is mainly driven by same-sex twins who work at the same firm, suggesting that social interactions may lead to an overestimate of the genetic component in earnings. Excluding siblings who work together leads to estimates of the heritability of log earnings that are 15% lower. Similar biases may be present in CTD-based estimates of heritability for other social outcomes like education.

## Significance

Why do families matter so much for the labor market success of their children? One explanation is shared hereditary factors; another is family resources and family-related preferences. We use data for twins to measure the share of the variation in earnings attributable to inherited genetic factors versus other shared family characteristics and purely idiosyncratic factors. Standard methods indicate substantial genetic heritability of earnings, and of both individual and firm premium components. But when pairs who work at the same firm are excluded, the heritability of the firm component falls substantially. This suggests caution in interpreting heritability estimates for socially determined outcomes, where a tendency of twins to influence each other can inflate measured heritability.

Social scientists have long been interested in the intergenerational transmission of economic status, as measured by occupation (1–4), earnings (5–7), or income (8, 9). Part of the correlation in economic status between parents and children is due to the effect of parents’ resources and social status. Part is also due to genetic inheritance. Typical data sources do not allow a clear distinction between these channels.

Twins studies offer a solution to this problem, making it possible to measure the heritability of physical traits like height (10) as well as of social outcomes like IQ (11), education (12), and income (13–16).^*^ Monozygotic (identical; MZ) twins share all of their genes, while dizygotic (fraternal; DZ) twins share the same fraction as other siblings. Both types of twin pairs share a family environment. In the standard ACE model—also known as the “classical twin design” or “CTD” (17)—the heritability of a trait, h^2^, defined as the share of the variance of the trait attributable to genes, is measured as twice the difference between the MZ and DZ correlations of that trait. A study of twins’ heights using data from seven countries (18) finds values of h^2^ from 0.68 to 0.85. A meta-analysis of twins’ height studies (18) reports a mean h^2^ of 0.77 for boys and 0.62 for girls at age 17. A broader meta-analysis of CTD studies (19) classifies 2,748 studies by outcome domain. Most of the studies relate to physical and mental health domains, but the authors identify 292 studies of cognitive traits (average h^2^ = 0.468; SE = 0.010), 56 studies of interpersonal interactions and relationships (average h^2^ = 0.319; SE = 0.038), and 35 of social values (average h^2^ = 0.313; SE = 0.019).

Unlike genomic studies that use direct measures of genotypes, and thus must rely on the selective samples and limited outcomes available in biobanks (e.g., ref. 20), twins studies do not use genetic information. Standard implementations do, however, rely on the distinction between MZ and DZ twin pairs—information that is usually unavailable in population data. As a result, most twin studies use twins who happen to be included in specialized surveys (e.g., the Add Health data). Samples are often very small.

We report twins correlations for log earnings based on the full population of twins from eight birth cohorts (born 1983–1990) in the United States (N = 44,500 twin pairs^†^). We use administrative earnings data that lack information on zygosity. We can, however, distinguish same-sex (SS) from opposite-sex (OS) twins. All OS twins are DZ, while SS twins are a mix of MZ and DZ, with estimable proportions. These data are sufficient to identify h^2^ under standard CTD assumptions.

If boys and girls are treated differently in some families, however, SS twin correlations will be higher than OS correlations, biasing estimates of h^2^ based on the standard ACE assumptions. We extend the model to allow for a family-by-sex component, which we identify by comparing SS to OS nontwin siblings. The SS–OS gap in sibling correlations is much larger for twins than for nontwin siblings—even those very close in age. In our extended ACE model, we identify h^2^ from the difference-in-differences of these correlations—SS minus OS twins vs. SS minus OS siblings. Using this approach, we estimate substantial heritability in the probability of employment (h^2^ = 0.23 to 0.32; SE = 0.05) and in log earnings conditional on working (h^2^ = 0.36; SE = 0.05).

There have been a few twins-based estimates of the heritability of labor market outcomes. Our samples are much larger and more representative than others in the existing literature (e.g., ref. 12). Nevertheless, our specific h^2^ estimates have to be interpreted carefully, as they rely on the assumptions of our extended ACE (E-ACE) model. Specifically, the model assumes that the family environment is equally shared by MZ and DZ twins, and by SS and OS twins, net of a gender difference that is the same for SS twins as for nontwin SS siblings but potentially different for girls and boys in the same family (12, 21–23). We present evidence in support of this assumption. Insofar as some of the shared component of earnings for identical twins is attributable to direct interactions between the twins, however, (e.g., ref. 24), our E-ACE model will overstate h^2^.

A burgeoning literature in labor economics decomposes log earnings into “person” and “firm” components, using what is known as the AKM model [(25); see also ref. 26]. The “person effect” in an AKM model is the component of earnings that remains constant as a person switches between jobs. It is often interpreted as reflecting a person’s human capital—broadly defined to include personality traits, ambition, etc. The “firm effect” is the earnings premium or discount that is shared by all workers at a given firm and is often interpreted as reflecting the firms’ pay policies. Neoclassical economic models of the labor market assume there are no firm-specific pay premiums. A growing body of studies, however, finds evidence of such premiums, accounting for 10 to 20% of the variation in wages (26).

Of great interest is understanding which workers obtain jobs at high-paying firms, and why (e.g., refs. 27–29). Does access to high-paying jobs represent pure luck (30), or is it related to worker skills or attributes? We use the AKM model to decompose earnings into individual and firm effects, and estimate the heritability of each component. We expect the heritability of earnings to be largely driven by heritability of the person component. Firm premiums could also be partly heritable, to the extent that firms that offer higher premiums tend to hire workers with high person effects (so-called “assortative matching”). Existing evidence on the correlation between person effects and firm premiums, however, suggest that this channel would lead to limited heritability in firm premiums—25% or less of the heritability of person effects.^‡^

Estimates from our E-ACE model imply strong heritability of person effects (h^2^ = 0.40; SE = 0.05) and moderate heritability of firm effects (h^2^ = 0.21; SE = 0.08). The latter is about 2× larger than could be explained by assortative matching alone. We find heritability of firm effects even within regions and industries, suggesting that it is not solely due to inherited preferences over geography or type of work. On its face, this seems to suggest that genetic factors affect not only workers’ skills but also their access to higher-paying firms.

Economic outcomes like earnings are different from physical outcomes like height or IQ, in that they depend on social interactions. For example, whether an individual takes a particular job depends on how much they value specific features of the job—such as who else works there. Social interactions could inflate the correlation of the firm effects of MZ twins relative to the correlation of siblings (31, 32). Suppose, for example, that identical twins have a stronger preference to work together than do same-sex siblings. Because people working at the same firm necessarily have the same firm effects, this will lead to an upward bias in the estimated heritability of firm-specific pay premiums from the E-ACE model. In our data, fully 11.4% of SS twin pairs are observed working for the same firm, nearly twice the rate for nontwin SS siblings (5.7%) and 3× or more the rate for OS twins (3.8%) or OS siblings (2.2%).

In the language of Manski (33), the earnings components of the ACE model are “correlated effects”—determinants of the outcomes of siblings that reflect the influence of unobserved but common genes or a common family environment. But there may also be what Manski identified as “endogenous effects,” where one child’s outcome directly influences that of his or her sibling. We suspect that the decision to work at the same firm as one’s twin could be driven in part by such endogenous social effects.

To address the potential biases in the components of our E-ACE model induced by endogenous interactions between twins, we re-estimate the model using a sample that excludes sibling pairs who work at the same firm. The estimated heritability of firm effects falls substantially and is no longer statistically distinguishable from zero (h^2^ = 0.07; SE = 0.08) or from a value that is one-quarter of the heritability of person effects (i.e., 0.35 ÷ 4 = 0.09). The estimated heritability of earnings as a whole also falls by about 15% (h^2^ = 0.31; SE = 0.08). This points to the potential importance of endogenous interactions among twins (e.g., choosing to work at the same job, or to attend the same college) that can lead to inflated estimates of the heritability of social outcomes from a CTD approach. Similar biases could arise in CTD studies of heritability of outcomes like education, which could also be influenced by interactions among twins. Such biases could be avoided in designs that use individual data on genotypes, such as polygenic index designs (34). Thus, our analysis points to a potential source of the “missing heritability” problem (35), whereby polygenic studies estimate lower heritability than do twin studies.

## Results

Our linked dataset, described in the methods section, includes 44,500 twin pairs with sufficient labor market attachment to measure wages. Of these, 31,500 are SS and 13,000 are OS. The data also include 507,000 SS sibling pairs and 500,000 OS sibling pairs. Among children (including nontwins) born in the same cohorts and observed at the same dates, 50.8% are male. We take that as an estimate of the fraction male among all births meeting the conditions for inclusion in our sample (including survival to adulthood and having sufficient labor market attachment). The implied share of SS twins who are MZ is s = 58.7%.

**Fig. 1.**
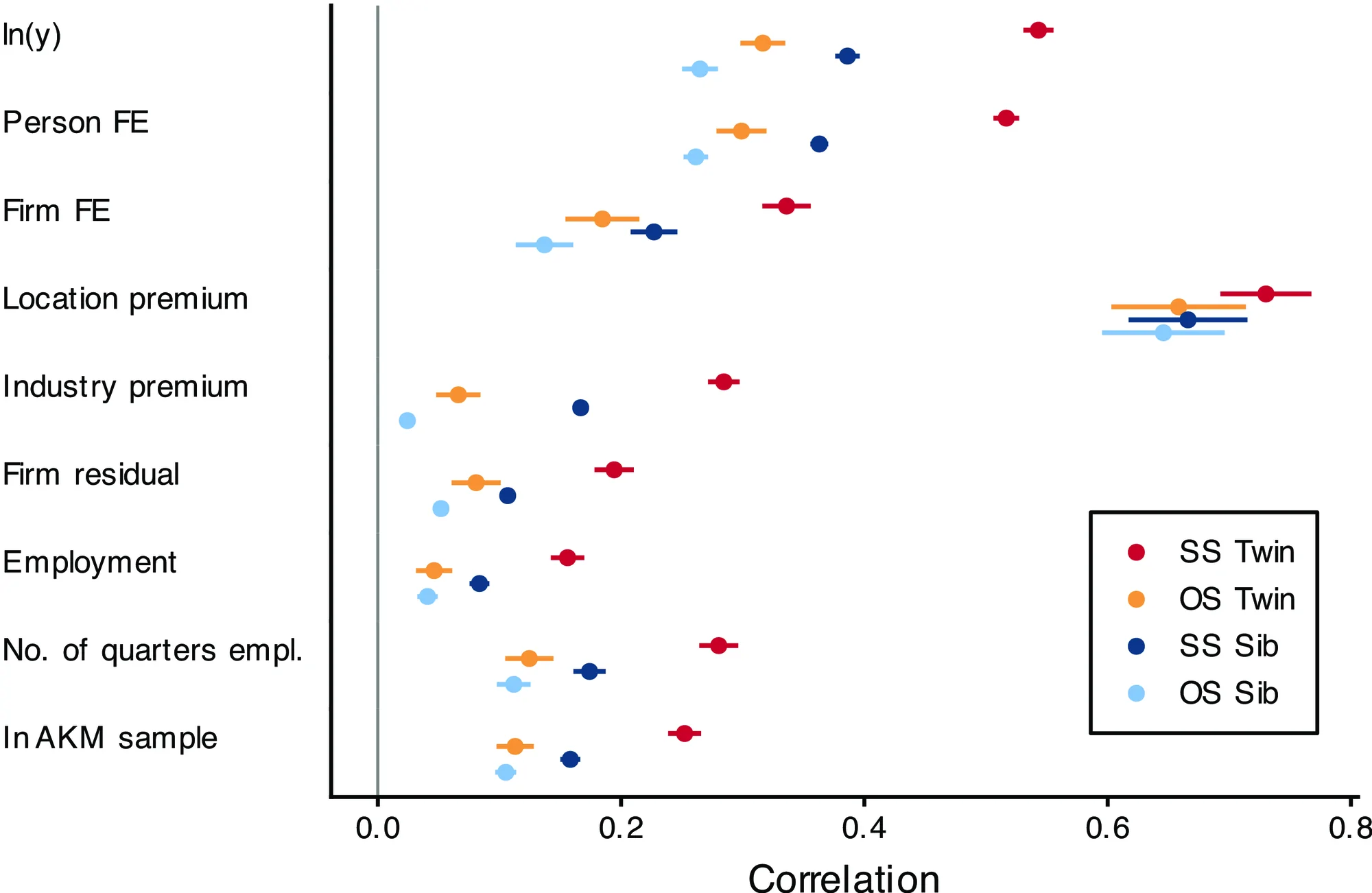
Between-sibling and between-twin correlations, same-sex (SS), and opposite-sex (OS). Horizontal lines represent 95% CIs. Log earnings and its components, presented in the first six panels, are computed over the sample of twin and sibling pairs who are both stably employed, as described in the data section. Employment outcomes, in the final three panels, are computed over all sibling pairs, including those who are not both stably employed.

Fig. 1 presents the correlations of various outcomes between SS twins ($\rho_{\mathrm{SS}}$), OS twins ($\rho_{\mathrm{OS}}$), SS siblings ($\rho_{\mathrm{SSsib}}$), and OS siblings ($\rho_{\mathrm{OSsib}}$). The first panel shows the correlations of log average quarterly earnings for the four groups. This is 0.543 (SE = 0.006) for SS twins, but much lower for the other comparisons. We note that the estimated correlation in earnings for same sex siblings—0.386 (SE = 0.005)—is very close to the average of the correlations for nontwin brothers (0.430) and nontwin sisters (0.323) reported in ref. 36. SS siblings have a stronger correlation of earnings than OS siblings, consistent with the presence of a gender-specific family factor in our E-ACE model, but the SS–OS difference is much larger for twins. The difference-in-differences is $\left(\left(\rho_{\mathrm{SS}}\;-\;\rho_{\mathrm{OS}}\right)\;-\;\left(\rho_{SSsib}\;-\;\rho_{OSsib}\right)\right)$ = 0.105 (SE = 0.014). Under the baseline assumption of our E-ACE model developed in the methods section—that siblings share one-half of their genes—this implies h^2^ = 0.36 (SE = 0.05).

The next two panels separate mean log earnings into person effect and firm effect components. In each case, $\rho_{\mathrm{SS}}$ is much larger than the other three correlations, and the difference-in-differences is positive—more so for the person effect (h^2^ = 0.40, SE = 0.04) than for the firm effect (h^2^ = 0.21, SE = 0.08). The following three panels decompose the firm effects into three components following (37): A location premium, defined as the average firm effect among all firms in the commuting zone; an industry premium, defined as the average firm effect among all firms in the industry; and a remainder. All three are correlated among twins and siblings, in each case more so for SS twins than for other pairs.

These estimates all condition on significant labor force attachment—pairs in which either sibling does not work (or only works infrequently) are excluded. In this regard the similarity of our estimate of the correlation in earnings between same-sex nontwin siblings with the estimates presented by (36), which are based on a broader sample, is reassuring. The remaining panels in Fig. 1 present sibling and twin correlations of three measures of employment status, estimated over the full population of sibling pairs. All show modest positive correlations among siblings, stronger for SS twins than for any of the other groups. We also investigated the stability of the estimated correlations in Fig. 1 across different ages (38) and found very similar estimates for siblings in the 8-y age range in our data.

**Fig. 2.**
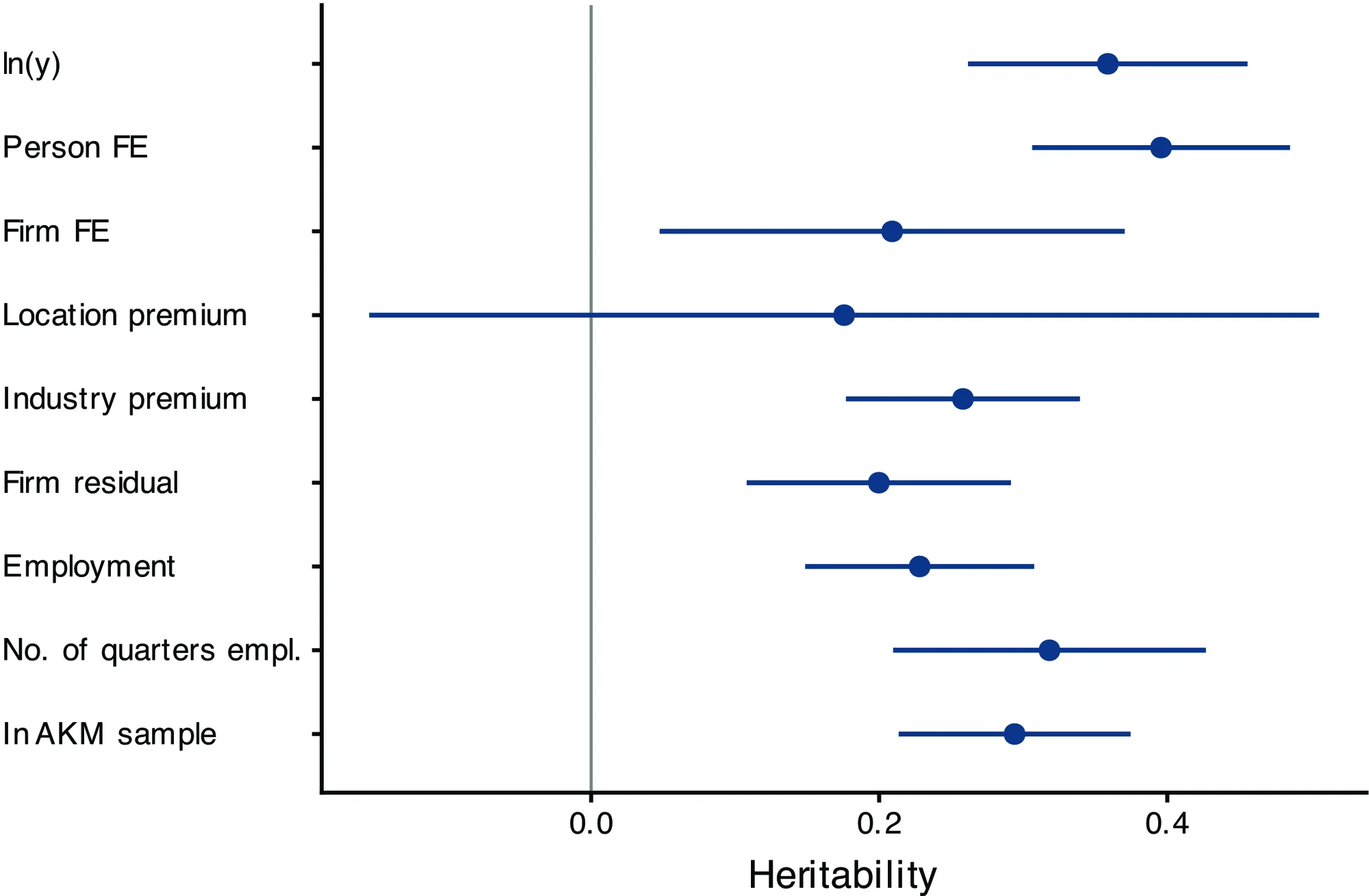
Heritability estimates of outcome noted on the y-axis from E-ACE model. Horizontal lines represent 95% CIs.

Table 1 presents the correlations plotted in Fig. 1, along with differences between SS and OS pairs, the difference-in-differences, and the implied heritability. All outcomes show positive heritability, with larger estimates for log earnings and the person effects than for the other components. All except the location premium, which is imprecisely estimated due to the large share of children who remain in the CZ where they grew up, are significantly different from zero. Heritability estimates are plotted in Fig. 2.

**Table 1.** Correlations of outcomes among twins and siblings and implied heritability estimates

	Correlations				Differences in correlations				
	Twins		Siblings						
	SS	OS	SS	OS	SS-OS twins	SS twins-SS siblings	SS-OS siblings	Difference in differences of correlations	Implied heritability (h^2^)
	(1)	(2)	(3)	(4)	(5)	(6)	(7)	(8)	(9)
Employment outcomes								
N (×1,000)	60	27	1,072	1,096					
Any employment	0.156 (0.007)	0.046 (0.007)	0.084 (0.004)	0.041 (0.004)	0.110 (0.010)	0.072 (0.008)	0.043 (0.006)	0.067 (0.012)	0.23 (0.04)
No. of quarters employed	0.280 (0.008)	0.125 (0.010)	0.174 (0.007)	0.112 (0.007)	0.156 (0.013)	0.106 (0.010)	0.062 (0.010)	0.093 (0.016)	0.32 (0.05)
In analysis sample	0.252 (0.007)	0.113 (0.008)	0.158 (0.004)	0.105 (0.004)	0.139 (0.010)	0.094 (0.008)	0.053 (0.006)	0.086 (0.012)	0.29 (0.04)
Earnings outcomes								
N (×1,000)	32	13	507	500					
Log earnings	0.543 (0.006)	0.317 (0.009)	0.386 (0.005)	0.265 (0.007)	0.227 (0.011)	0.157 (0.008)	0.121 (0.009)	0.105 (0.014)	0.36 (0.05)
AKM person effect	0.517 (0.005)	0.299 (0.010)	0.363 (0.004)	0.261 (0.005)	0.218 (0.012)	0.154 (0.006)	0.102 (0.006)	0.116 (0.013)	0.40 (0.04)
AKM firm effect	0.336 (0.010)	0.185 (0.015)	0.227 (0.010)	0.137 (0.012)	0.151 (0.018)	0.109 (0.014)	0.090 (0.015)	0.061 (0.024)	0.21 (0.08)
Location premium	0.730 (0.019)	0.658 (0.028)	0.666 (0.024)	0.646 (0.025)	0.072 (0.033)	0.064 (0.031)	0.020 (0.035)	0.052 (0.048)	0.18 (0.16)
Industry premium	0.284 (0.006)	0.066 (0.009)	0.167 (0.003)	0.024 (0.003)	0.218 (0.011)	0.118 (0.007)	0.142 (0.004)	0.076 (0.012)	0.26 (0.04)
Firm residual premium	0.194 (0.008)	0.081 (0.010)	0.107 (0.003)	0.052 (0.003)	0.113 (0.013)	0.088 (0.008)	0.055 (0.004)	0.059 (0.013)	0.20 (0.05)

Notes: SS = same sex; OS = opposite sex. See text for definitions of AKM effects and subcomponents of the AKM firm effect. SE in parentheses. Correlation SE are computed from the regression of one sibling’s outcome on the other’s, with robust SE. SE for differences, difference-in-differences, and heritability statistics are computed from the correlation SE using the delta method.

**Fig. 3.**
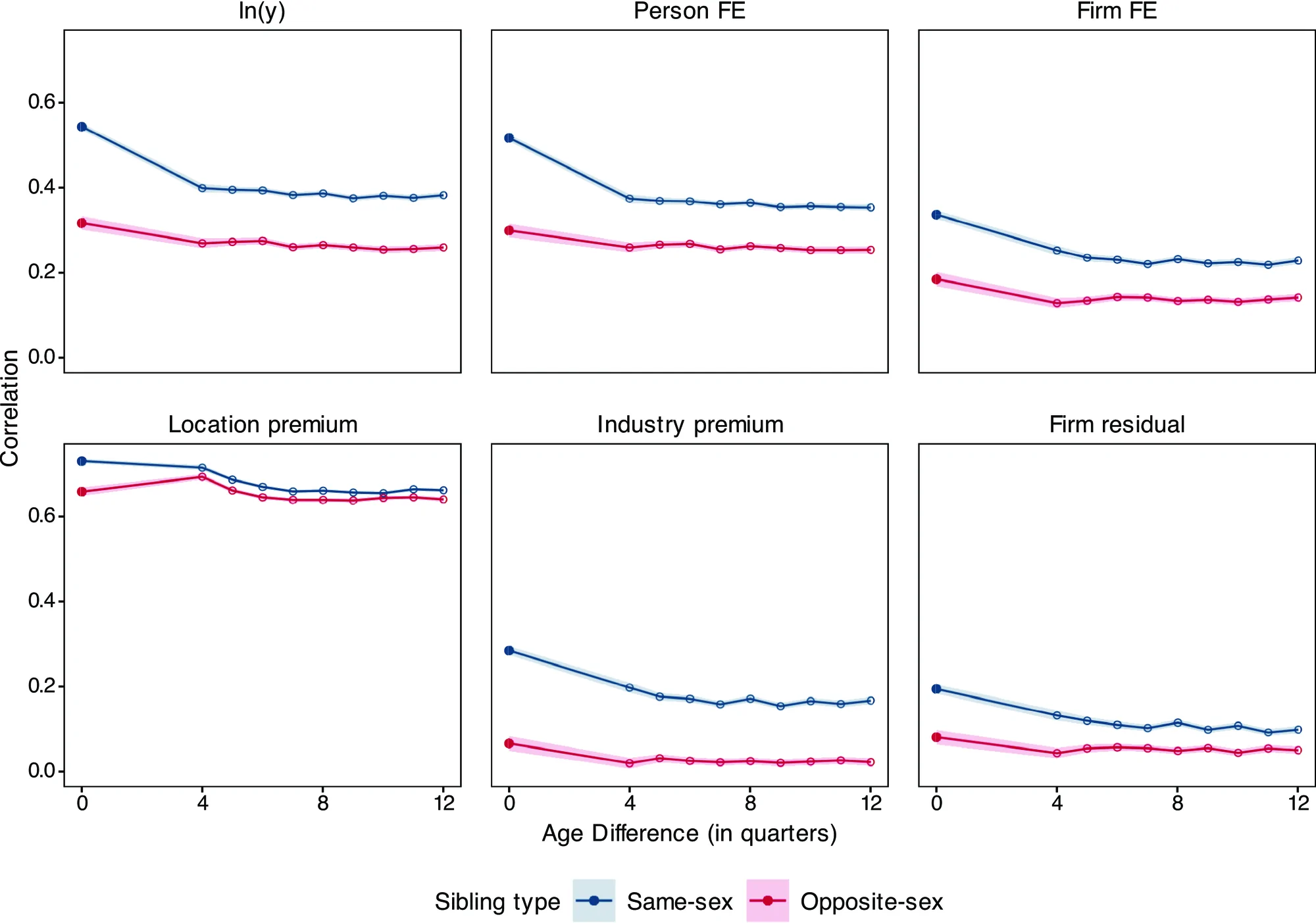
Between-sibling correlations, by age difference between siblings. The shaded region indicates 95% pointwise CI. This is computed using traditional, homoskedastic formula for the SE of a correlation coefficient.

Fig. 3 presents correlations computed over subsamples of siblings separated by different gaps in age, ranging from 1 y to 3 y. Among nontwin siblings, the correlations decay only slightly as the age gap grows. This suggests that common family environmental influences on earnings (the C component of the ACE model) do not fade dramatically as the age gap between siblings rises. Moreover, the SS and OS correlations decay at similar rates, supporting our modeling assumption that there is a stable family-by-gender component in earnings. We do not reject the hypothesis that the SS–OS difference in log earnings correlations is equal across all age gaps from 4 to 12 quarters (χ^2^ = 4.21, d.f. = 8, *P* = 0.84).

When we consider twins (shown in Fig. 3 with an age gap of 0), we see higher SS correlations for nearly all outcomes than for SS siblings who are even 1 y apart. In contrast, the difference in correlations between OS twins and OS siblings is very small, as predicted by our E-ACE model. This buttresses our interpretation that the difference-in-differences filters out both the overall family component in earnings and any family-by-gender component in the $\left(\rho_{\mathrm{SS}}\;-\;\rho_{\mathrm{OS}}\right)$ gap, isolating the component due to differences in the genetic similarity of SS vs. OS twins. We note, however, that if the family-by-gender component affecting same sex twins is larger than that affecting same sex siblings even 1 y apart, our approach will overstate heritability.

Table 2 presents estimates of h^2^ for two samples. The first column repeats results for our main sample from Table 1. Column 2 excludes twin and sibling pairs who work at the same firm. The heritability estimate for the AKM person effect falls by about 15% while the estimate for the firm effect falls by two-thirds and is no longer statistically significant (h^2^ = 0.07, SE = 0.08). The relative size of the heritability shares of the two earnings components in column 2 is in the range that would be predicted if *all* the genetic influence on the firm component was driven by the tendency of higher-paying firms to hire workers with higher person effects.

**Table 2.** Sensitivity of estimated heritability to firm and industry overlap

	Estimated heritability	
	Full sample	No firm overlap sample
	(1)	(2)
Log earnings	0.36 (0.05)	0.31 (0.05)
AKM person effect	0.40 (0.04)	0.35 (0.05)
AKM firm effect	0.21 (0.08)	0.07 (0.08)
Location premium	0.18 (0.16)	0.13 (0.17)
Industry premium	0.26 (0.04)	0.12 (0.04)
Firm residual premium	0.20 (0.05)	-0.01 (0.04)
Number of twin pairs (×1,000)	45	41
Fraction in same firm	9%	0%
Number of sibling pairs (×1,000)	1,007	967
Fraction in same firm	4%	0%

Notes: See note to Table 1. “No firm overlap” sample excludes twin or sibling pairs for which there is any overlap in the list of firms at which the two siblings worked in 2018-2023Q1. SE in parentheses.

## Discussion

Interpretation of CTD estimates is complex, especially for social outcomes. The estimated heritability of outcomes may be biased in the presence of assortative mating, genetic dominance, population stratification, or violations of the assumption that the home environment is shared to the same degree for MZ as for DZ twins. Moreover, even when estimated without bias, heritability need not indicate direct genetic effects—it also may reflect indirect or cultural effects (e.g., effects of the parents’ genes on a child’s outcomes, not operating through the child’s own genes; see, e.g., refs. 22, 39, and 40). In the case of outcomes like earnings another channel arises that may not be relevant for a purely physical outcome like height: Twins may influence each other directly—for example, in their choice of jobs—inflating the correlation in their outcomes.

Using our overall dataset of twins and siblings we find that labor market outcomes are heritable, with estimated heritability of 0.40 for the person effect component of earnings and 0.36 for earnings as a whole. Heritability of the firm effect component is somewhat smaller (0.21) but still surprisingly high. When we exclude sibling pairs working at the same firm to limit the effect of social interactions between twins, however, the estimated heritability of the firm component falls substantially and is no longer statistically significant.

There are nongenetic reasons for twins to tend to work at the same firm. Thus, we interpret the estimates excluding same-firm pairs as the best estimates of heritability. These indicate that log wages and the person effect component are strongly heritable (h^2^ = 0.31 and 0.35 respectively) but that the firm effect (h^2^ = 0.07) is not. The fall in the heritability of firm effects when twins who work for the same firm are excluded also raises a caution about potential confounding of the CTD when applied to other outcomes where individual choice plays a role. One can easily imagine similar mechanisms affecting the education choices of SS twins, arising from a desire to stay together rather than from any shared genetic factors. Such interactions could inflate the correlations in the person effects of same sex twins, implying that even our estimates derived from the sample excluding siblings who work together may be inflated by unobserved social interaction effects.

## Materials and Methods

### Data.

This study uses deidentified records maintained at the US Census Bureau.^§^ All individual identifiers were removed prior to access by the researchers and replaced by protected identification keys (PIKs). All results were reviewed under the US Census Bureau’s Disclosure Review policy to ensure that the risk that disclosed results could be used to identify an individual is very small. Consequently, the research does not qualify as human subjects research.

Our analysis is based on children identified in the US 2000 population census who were the biological child of the household head. We identify twins as children in the same household with the same birthdate (±2 d), and siblings as children with different birthdates (9 mo or more apart). The US Census Bureau assigns Personal Identification Keys (PIKs) to all respondents using name and birthdate information. We limit attention to twin or sibling pairs in which both children were assigned PIKs and were between 9 and 17 y old at the time of the census. Families with three or more children can contribute multiple pairs to the sample.

These individuals are linked to the Longitudinal Employer Household Dynamics (LEHD) data, which reports the quarterly earnings received by employees from each employer. LEHD data are derived from unemployment insurance tax payments, and cover all formal sector wage and salary jobs (i.e., excluding self-employment) apart from the federal government. We use LEHD data from 2018 to the first quarter of 2023, excluding the second, third, and fourth quarters of 2020 when the labor market was substantially disrupted by the COVID pandemic. We focus on individuals who are observed in the LEHD in this period with at least eight quarters of observed earnings above $3,800 (approximately the minimum wage for full-time work), excluding the first and last quarter of each employment spell and any quarter with multiple employers. We interpret observed quarterly earnings for this sample as reflecting full-quarter, full-time earnings. In our analysis of earnings outcomes, we retain only sibling pairs in which both siblings meet the restrictions. Our primary outcome is average log earnings for the individual across all quarters meeting the above restrictions.

We also use three measures of employment: an indicator for having any employment during the sample period; the number of quarters with positive employment, and an indicator for having sufficient employment to enter into our sample. These are measured over the full population of sibling pairs, without conditioning on earnings.

We classify two siblings as working at the same firm if they had at least one employer in common in our 2018–2023 data window.^¶^ When we examine correlations for siblings observed with different age gaps, we exclude nontwin sibling pairs with gaps smaller than 1 y, as these likely reflect adoptions or measurement error rather than actual sibling pairs.

### Extended ACE Model.

Let ${\mathrm{cov}}_{g}$ represent the covariance of long-term outcome $Y_{i}$ between siblings of type g, where the types to be considered are MZ twins (g=MZ); same-sex DZ twins (g=DZSS); all same-sex twins, combining MZ and DZSS (g=SS); opposite-sex twins, who are all DZ (g=OS); same-sex, nontwin siblings (g=SSsib); and opposite-sex, nontwin siblings (g=OSsib). We can measure ${\mathrm{cov}}_{\mathrm{SS}}$, ${\mathrm{cov}}_{\mathrm{OS}}$, ${\mathrm{cov}}_{\mathrm{SSsib}}$, and ${\mathrm{cov}}_{\mathrm{OSsib}}$ directly.

In the basic ACE model (e.g., ref. 17), $Y_{i}$ is the sum of three factors: genetic endowment $A_{i}$, shared family environment $C_{f(i)}$, where f(i) is an index function pointing to the family of person i, and nonshared environment, $E_{i}$: $Y_{i}=A_{i}+C_{f\left(i\right)}+E_{i}$. The heritability of the outcome is defined as $h^{2}=V\left(A\right)/V\left(Y\right)$, the share of the variation that is explained by the genetic endowment.

MZ twins *i* and *j* are assumed to have identical genetic endowment $A_{i}=A_{j}$. DZ twins are assumed to have correlated genetic endowment $R_{A}=corr\left(A_{i},A_{j}\right)$, with $R_{A}<1$. As in other CTD studies, we assume $R_{A}=0.5$, as would arise under random mating, while acknowledging that with assortative mating $R_{A}$ might be larger.

Both MZ and DZ twins have identical shared environments, $C_{f(i)}=C_{f\left(j\right)}$, but nonshared environments that are assumed to be independently distributed across the relevant population. Assuming variances and the A to C covariance are the same for MZ and DZ twins, ${\mathrm{cov}}_{\mathrm{MZ}}=V\left(A\right)+V\left(C\right)+2cov\left(A,C\right)$ while ${\mathrm{cov}}_{\mathrm{DZ}}=R_{A}V\left(A\right)+V\left(C\right)+2cov\left(A,C\right)$. Thus, heritability can be estimated as $h^{2}=\frac{cov_{\mathrm{MZ}}-cov_{\mathrm{DZ}}}{\left(1-R_{A}\right)V\left(Y\right)}=2\left(\rho_{\mathrm{MZ}}-\rho_{\mathrm{DZ}}\right)$ when $R_{A}=0.5$.

We cannot distinguish MZ from DZSS twins, only SS from OS. The SS twin covariance is

$${\mathrm{cov}}_{\mathrm{SS}}\;=\;\left(s\;+\;\left(1\;-\;s\right)R_{A}\right)V\left(A\right)\;+\;V\left(C\right)\;+\;2cov(A,C),$$

where s≡p(MZ|SS) is the share of SS twins who are MZ. The OS twin covariance is the same as the DZ covariance,

$${\mathrm{cov}}_{\mathrm{OS}}\;=\;R_{A}V\left(A\right)\;+\;V\left(C\right)\;+\;2cov(A,C).$$

This implies $h^{2}=\left(cov_{\mathrm{SS}}-cov_{\mathrm{OS}}\right)/s\left(1-R_{A}\right)V\left(Y\right)$. Thus, the CTD can be extended directly to data where zygosity is not identified, so long as an estimate of s is available. We discuss estimation of s below.

We do not believe the simple SS–OS difference can be used to identify $h^{2}$ for labor market outcomes, however, as we expect the family environment relevant for labor market outcomes may differ for boys and girls in the same family. Some families are gender egalitarian, promoting sons’ and daughters’ labor market success to a similar degree, while others take more traditional views of gender roles, and may promote sons’ success more than daughters’. This would violate the equal environments assumption and reduce the OS correlation relative to the SS correlation.

We extend the ACE model by adding a second family environment term, G, that varies by child’s sex $g\left(i\right)$: $Y_{i}=A_{i}+C_{f\left(i\right)}+G_{f\left(i\right)g\left(i\right)}+E_{i}$. We assume that for two siblings *i* and *j* in the same family f, $corr\left(G_{fg\left(i\right)},G_{fg\left(j\right)}\right)=1$ if $g\left(i\right)=g\left(j\right)$ but $corr\left(G_{fg\left(i\right)},G_{fg\left(j\right)}\right)=\tau <1$ if $g\left(i\right)\neq g\left(j\right)$. For simplicity, we assume $cov\left(A,G\right)=cov\left(C,G\right)=0$, though this is not essential. In this extended model:

$${\mathrm{cov}}_{\mathrm{MZ}}\;=\;V\left(A\right)\;+\;V\left(C\right)\;+\;V\left(G\right)\;+\;2cov\left(A,C\right),$$

$${\mathrm{cov}}_{\mathrm{DZSS}}\;=\;R_{A}V\left(A\right)\;+\;V\left(C\right)\;+\;V\left(G\right)\;+\;2cov\left(A,C\right),$$

$${\mathrm{cov}}_{\mathrm{SS}}\;=\;\left(s\;+\;\left(1\;-\;s\right)R_{A}\right)V\left(A\right)\;+\;V\left(C\right)\;+\;V\left(G\right)\;+\;2cov\left(A,C\right),$$

$${\mathrm{cov}}_{\mathrm{OS}}\;=\;R_{A}V\left(A\right)\;+\;V\left(C\right)\;+\;\tau V\left(G\right)\;+\;2cov\left(A,C\right).$$

In the presence of a family component that varies by sex, we cannot isolate $V\left(A\right)$ based only on the difference in covariances between SS and OS twins, since:

$${\mathrm{cov}}_{\mathrm{SS}}\;-\;cov_{\mathrm{OS}}\;=\;s\left(1\;-\;R_{A}\right)V(A)\;+\;\left(1\;-\;\tau \right)V(G).$$

To proceed, we augment this system with covariances for nontwin siblings. We assume the G correlations are the same as for twins: 1 if same-sex and τ if opposite sex. But the common family environment effect C may differ for siblings, whose time in the home only partly overlaps. We assume that the correlation of this component is θ<1 for nontwin siblings. We further assume that A is correlated the same for siblings as for DZ twins, $R_{A}$. This implies

$${\mathrm{cov}}_{\mathrm{SSsib}}\;=\;R_{A}V\left(A\right)\;+\;\theta V\left(C\right)\;+\;V\left(G\right)\;+\;2cov\left(A,C\right),$$

$${\mathrm{cov}}_{\mathrm{OSsib}}\;=\;R_{A}V\left(A\right)\;+\;\theta V\left(C\right)\;+\;\tau V\left(G\right)\;+\;2cov\left(A,C\right),$$

$${\mathrm{cov}}_{\mathrm{SSsib}}\;-\;{\mathrm{cov}}_{\mathrm{OSsib}}\;=\;\left(1\;-\;\tau \right)V(G).$$

Combining the expressions for the differences in covariances between same-sex and opposite sex twins versus siblings, we obtain:

$$h^{2}\;=\;\frac{\left(cov_{\mathrm{SS}}\;-\;cov_{\mathrm{OS}}\right)\;-\;\left({\mathrm{cov}}_{\mathrm{SSsib}}\;-\;{\mathrm{cov}}_{\mathrm{OSsib}}\right)}{s\left(1\;-\;R_{A}\right)V\left(A\right)}.$$

The numerator of this expression is the difference in differences in covariances between same sex and opposite sex twins vs. siblings. The denominator is a scale factor that reflects the fraction of MZ’s among same sex twins (*s*) and the share of the genetic component shared by DZ twins ($R_{A}$). Note that this expression does not depend on the specific value of θ.

We assume $R_{A}=0.5$ for the estimates presented in the text. Existing research suggests that assortative mating may lead to a positive correlation between husbands and wives in the components of genes that are related to education, suggesting a higher value of $R_{A}$. For example, a recent study (41) reports a correlation of 0.14 in the polygenic scores for education between spouses. The same correlation in the genetic components of earnings would imply $R_{A}=0.57$, leading to estimates of $h^{2}$ that are 16% above our reported values. We also assume that the variances of all elements of our extended ACE model are constant across types of siblings (twins vs. not, SS vs. OS), and report estimated correlations and the value $h^{2}$ rather than V(A).

To estimate $s\equiv p\left(MZ|SS\right)$, we first use the excess fraction of SS twins above the rate for DZ twins to estimate the share of MZ twins:

$$p\left(\mathrm{MZ}\right)\;=\;\frac{p\left(SS\right)\;-\;p\left(SS\;|\;DZ\right)}{1\;-\;p(SS\;|DZ)}.$$

Assuming that the sexes of DZ twins are determined independently with probability π of being male (which we estimate at 0.508), we get $p\left(SS|DZ\right)=\pi^{2}+$${\left(1-\pi \right)}^{2}=0.500$. With $p\left(SS\right)=0.708$ (in our sample), we estimate $p\left(MZ\right)=$0.416 and $p\left(MZ|SS\right)=0.588$.

A key assumption of our E-ACE model is that the gender-specific component of family environment (G) is perfectly correlated across siblings and additively separable from the nongendered component. This allows us to remove the bias in ${\mathrm{cov}}_{\mathrm{SS}}-cov_{\mathrm{OS}}$ by subtracting off the analogous difference for siblings. A second assumption is that the correlation between the shared family component (C) in sibling outcomes is invariant to the age gap between siblings. If instead the correlation decays with the age gap, but at the same rate for SS and OS siblings, then our approach remains valid providing the distributions of age gaps are similar for SS and OS siblings. The parallel and nearly flat profiles of the correlations in log earnings for SS and OS siblings at different age gaps shown in Fig. 3 suggest that the decay rates of the sibling correlations are similar for SS and OS siblings and very small.

### SE.

We use a heteroskedasticity-robust regression-based approach to compute the SE of our correlations. If $\beta_{\mathrm{XY}}$ is the coefficient of a regression of *Y* on *X*, it is related to the correlation coefficient by $\rho_{\mathrm{XY}}=\frac{\sigma_{X}}{\sigma_{Y}}\beta_{\mathrm{XY}}$, where $\sigma_{Y}$ and $\sigma_{X}$ are the SD of *Y* and *X*, respectively. We thus compute the sampling variance of the correlation coefficient as $V\left(\rho_{\mathrm{XY}}\right)=\frac{\sigma_{X}^{2}}{\sigma_{Y}^{2}}V\left(\beta_{\mathrm{XY}}\right)$, where $V(\beta_{\mathrm{XY}})$ is the heteroskedasticity-robust variance of $\beta_{\mathrm{XY}}$ and we treat the variances of *X* and Y as known. This yields slightly larger SE than the traditional formula for the SE of an estimated correlation, $\frac{1-\rho^{2}}{\surd N}$.

Our estimate of $h^{2}$ is computed from the difference-in-differences of correlation coefficients from independent samples, multiplied by a constant $\frac{1}{s\left(1-R_{A}\right)}$. We treat $s\left(1-R_{A}\right)$ as known. Thus, the variance of $h^{2}$ is the sum of the variances of the underlying correlations, multiplied by the square of the constant.

### Decomposing Log Earnings Using the AKM Model.

We use a decomposition based on ref. 25 to decompose quarterly earnings in the LEHD file. In brief, the model can be written as

$$y_{\mathrm{it}}\;=\;\alpha_{i}\;+\;\psi_{j\left(i,t\right)}\;+\;X_{\mathrm{it}}\beta \;+\;\xi_{\mathrm{it}},$$

where $y_{\mathrm{it}}$ represents the log earnings of individual i in quarter t, $\alpha_{i}$ is a person fixed effect, $\psi_{j\left(i,t\right)}$ is a fixed effect for the establishment at which the person works in quarter t, $j\left(i,t\right)$ is an index function giving the workplace of individual i in quarter t, $X_{\mathrm{it}}\beta$ captures effects of time-varying observable covariates, such as experience and calendar time, and $\xi_{\mathrm{it}}$ is assumed to be orthogonal to the sequence of establishment effects, conditional on $\alpha_{i}$ (see ref. 42). In the AKM literature $\alpha_{i}$ is interpreted as an individual’s earnings capacity at any job, while $\psi_{j}$ is interpreted as a wage premium or discount at establishment j relative to pay in a reference workplace (which we take to be an average employer in the restaurant sector).

We estimate this model using data on all individuals in the LEHD from 2018Q1 to 2023Q1, omitting data from 2020Q2 through 2020Q4 during the COVID epidemic. The sample is limited to people who have at least eight quarters with earnings over $3,800. We use only quarters when an individual is between 20 and 60 y of age and has a single employer. The first and last quarters on a given job are also excluded. After model estimation, we assign the estimated person effects to the individuals who are either twins or siblings in our analysis sample. Similarly, we assign the mean of the estimated pay premiums for firms at which they worked in all the quarters they are observed satisfying the restrictions for our estimation sample. The sum of these two components accounts for 82% of the variance in person-quarter log earnings in the cohorts from which our twin sample is drawn (28). We further decompose the firm pay premiums into location, industry, and residual components, following (37).

We note that the AKM model makes the strong assumption that the person and firm components of wages multiply together, so the logarithm of earnings is additive in the two parts. Violation of this assumption could lead to problems in interpreting our decomposition. Fortunately, tests reported by refs. 42, 43, and 44 and summarized in ref. 26 suggest that violations of additivity in the logarithm of earnings, while present, are relatively minor.

## Data Availability

Some study data are available. The data are from confidential data available at the US Census Bureau. Researchers may apply for access to the Census Bureau microdata used in this study through the Federal Statistical Research Data Center (FSRDC) program, subject to Census Bureau approval and confidentiality requirements. The exact internal LEHD files used in this study are available only to Census Bureau internal researchers and are not part of the FSRDC-curated data; however, related LEHD and linked Census microdata are available to researchers through approved FSRDC projects. An archive that includes the programs used to construct the estimates in this paper is available at ref. 45.

## Footnotes

^*^A separate social sciences literature uses differences between siblings or twins to identify the causal return to education, on the grounds that twins have identical ability and thus differences between them remove omitted variables bias (46–48). Other literatures use adoptees (e.g., refs. 16, 49, and 50) or direct genetic measures (see the review in ref. 34), rather than twins, to identify heritability.

^†^Our analyses rely on confidential data maintained by the US Census Bureau. Sample sizes are rounded to the nearest 100.

^‡^Existing studies find that the correlation of worker and firm effects in earnings in the U.S. labor market is 0.50 or less (see e.g., ref. 43). This implies that the part of the firm effect that is attributable to the worker abilities and traits embedded in the person effect explain 25% or less of the variance in firm premiums.

^§^See Data, Materials and Software Availability statement for information on access to these data.

^¶^The LEHD assigns workers to specific establishments rather than to firms (e.g., McDonalds). All references to “firms” in fact refer to specific establishments. Note that with our definition siblings may be counted as working at the same firm even if their employment spells did not overlap.

## References

[1] P. M. Blau, O. D. Duncan, The American Occupational Structure (Wiley, New York, 1967).

[2] C. Jencks , Who Gets Ahead? The Determinants of Economic Success in America (Basic Books, New York, 1979).

[3] J. H. Goldthorpe, C. Llewellyn, C. Payne, Social Mobility and Class Structure in Modern Britain (Clarendon Press, Oxford, 1980).

[4] M. Hout, More universalism, less structural mobility: The American occupational structure in the 1980s. Am. J. Sociol. **93**, 1358–1400 (1988).

[5] J. Behrman, P. Taubman, Intergenerational earnings mobility in the United States: Some estimates and a test of Becker’s intergenerational endowments model. Rev. Econ. Stat. **67**, 144–151 (1985).

[6] G. S. Becker, N. Tomes, Human capital and the rise and fall of families. J. Labor Econ. **4**, S1–S39 (1986). 10.1086/298118 12146356

[7] D. J. Zimmerman, Regression toward mediocrity in economic stature. Am. Econ. Rev. **82**, 409–429 (1992).

[8] D. J. Treiman, R. M. Hauser, “Intergenerational transmission of income: An exercise in theory construction” in The Process of Stratification: Trends and Analyses, R. M. Hauser, D. L. Featherman, Eds. (Academic Press, New York, 1977), pp. 271–302.

[9] G. Solon, Intergenerational income mobility in the United States. Am. Econ. Rev. **82**, 393–408 (1992).

[10] K. Silventoinen , Heritability of adult body height: A comparative study of twin cohorts in eight countries. Twin Res. **6**, 399–408 (2003). 14624724 10.1375/136905203770326402

[11] R. Plomin, M. J. Owen, P. McGuffin, The genetic basis of complex human behaviors. Science **264**, 1733–1739 (1994). 8209254 10.1126/science.8209254

[12] P. Bingley, L. Cappellari, K. Tatsiramos, On the Origins of Socioeconomic Inequalities: Evidence from Twin Families (Luxembourg Institute of Socio-Economic Research, 2024).

[13] P. Taubman, The determinants of earnings: Genetics, family, and other environments; A study of white male twins. Am. Econ. Rev. **66**, 858–870 (1976).

[14] A. Björklund, M. Jäntti, G. Solon, “Influences of nature and nurture on earnings variation: A report on a study of various sibling types in Sweden” in Unequal Chances: Family Background and Economic Success, S. Bowles, H. Gintis, M. Osborne Groves, Eds. (Princeton University Press, Princeton, NJ, 2005), pp. 145–164.

[15] D. J. Benjamin , The promises and pitfalls of genoeconomics. Annu. Rev. Econ. **4**, 627–662 (2012). 10.1146/annurev-economics-080511-110939 PMC3592970 23482589

[16] B. Sacerdote, “Nature and nurture effects on children’s outcomes: What have we learned from studies of twins and adoptees?” in Handbook of Social Economics, J. Benhabib, A. Bisin, M. O. Jackson, Eds. (North-Holland, Amsterdam, The Netherlands, 2011), **vol. 1**, pp. 1–30.

[17] P. M. Visscher, Power of the classical twin design revisited. Twin Res. **7**, 505–512 (2004). 15527666 10.1375/1369052042335250

[18] R. Dewau , Meta-analysis of the heritability of childhood height from 560,000 pairs of relatives born between 1929 and 2004. Am. J. Hum. Biol. **37**, e24188 (2025). 39564936 10.1002/ajhb.24188

[19] T. J. C. Polderman , Meta-analysis of the heritability of human traits based on fifty years of twin studies. Nat. Genet. **47**, 702–709 (2015). 25985137 10.1038/ng.3285

[20] T. Schoeler , Participation bias in the UK Biobank distorts genetic associations and downstream analyses. Nat. Hum. Behav. **7**, 1216–1227 (2023). 37106081 10.1038/s41562-023-01579-9 PMC10365993

[21] S. Gusev, Twin heritability models can tell you whatever you want to hear. The Infinitesimal (2024). https://theinfinitesimal.substack.com/p/twin-heritability-models-can-tell. (Accessed 01 July 2026).

[22] S. Gusev, The missing heritability question is now (mostly) answered. The Infinitesimal (2025). https://theinfinitesimal.substack.com/p/the-missing-heritability-question. (Accessed 01 July 2026).

[23] C. H. Burt, R. L. Simons, Pulling back the curtain on heritability studies: Biosocial criminology in the postgenomic era. Criminology **52**, 223–262 (2014).

[24] J. Kaprio, M. Koskenvuo, R. J. Rose, Change in cohabitation and intrapair similarity of monozygotic (MZ) cotwins for alcohol use, extraversion, and neuroticism. Behav. Genet. **20**, 265–276 (1990). 2353911 10.1007/BF01067794

[25] J. M. Abowd, F. Kramarz, D. N. Margolis, High wage workers and high wage firms. Econometrica **67**, 251–333 (1999).

[26] P. Kline, “Firm wage effects” in Handbook of Labor Economics, C. Dustmann, T. Lemieux, Eds. (North-Holland, Amsterdam, The Netherlands, 2024), **vol. 5**, pp. 115–181.

[27] D. Card, J. Rothstein, M. Yi, Reassessing the spatial mismatch hypothesis. AEA Pap. Proc. **114**, 221–225 (2024).

[28] D. Card, J. Rothstein, M. Yi, “How do neighborhoods and firms affect intergenerational mobility?” Working paper. (University of California, Berkeley, CA, 2026). https://jesse-rothstein.com/wp-content/uploads/2026/02/CRY_IGE_Mar2026.pdf (Accessed July 1, 2026).

[29] F. Gerard, L. Lagos, E. Severnini, D. Card, Assortative matching or exclusionary hiring? The impact of employment and pay policies on racial wage differences in Brazil. Am. Econ. Rev. **111**, 3418–3457 (2021).

[30] G. S. Becker, N. Tomes, An equilibrium theory of the distribution of income and intergenerational mobility. J. Polit. Econ. **87**, 1153–1189 (1979).

[31] A. Horwitz , Rethinking twins and environments: Possible social sources for assumed genetic influences in twin research. J. Health Soc. Behav. **44**, 111–129 (2003). 12866384

[32] A. Stenberg, “Nature or nurture? A note on the misinterpreted twin decomposition”. Working paper 4/2011. (Swedish Institute for Social Research (SOFI), Stockholm, 2011).

[33] C. F. Manski, Identification of endogenous social effects: The reflection problem. Rev. Econ. Stud. **60**, 531–542 (1993).

[34] D. J. Benjamin, D. Cesarini, P. Turley, A. S. Young, Social-Science Genomics: Progress, Challenges, and Future Directions (National Bureau of Economic Research, 2024).

[35] B. Maher, Personal genomes: The case of the missing heritability. Nature **456**, 18–21 (2008). 18987709 10.1038/456018a

[36] B. Mazumder, Sibling similarities and economic inequality in the US. J. Popul. Econ. **21**, 685–701 (2008).

[37] D. Card, J. Rothstein, M. Yi, Industry wage differentials: A firm-based approach. J. Labor Econ. **42**, S11–S59 (2024).

[38] P. Bingley, L. Cappellari, Correlation of brothers’ earnings and intergenerational transmission. Rev. Econ. Stat. **101**, 370–383 (2019).

[39] S. Gusev, No, intelligence is not like height. The Infinitesimal (2024). https://theinfinitesimal.substack.com/p/no-intelligence-is-not-like-height. (Accessed 01 July 2026).

[40] L. J. Howe , Within-sibship genome-wide association analyses decrease bias in estimates of direct genetic effects. Nat. Genet. **54**, 581–592 (2022). 35534559 10.1038/s41588-022-01062-7 PMC9110300

[41] D. Hugh-Jones, K. J. H. Verweij, B. St. Pourcain, A. Abdellaoui, Assortative mating on educational attainment leads to genetic spousal resemblance for polygenic scores. Intelligence **59**, 103–108 (2016).

[42] D. Card, J. Heining, P. Kline, Workplace heterogeneity and the rise of West German wage inequality. Q. J. Econ. **128**, 967–1015 (2013).

[43] M. Lachowska, A. Mas, R. Saggio, S. A. Woodbury, Work hours mismatch. Econometrica **94**, 991–1025 (2026).

[44] D. Card, J. Rothstein, M. Yi, Location, location, location. Am. Econ. J. Appl. Econ. **17**, 297–336 (2025).

[45] D. Card, J. Rothstein, M. Yi, “Replication Code for: Heritability and social interaction in labor market outcomes: Evidence from the population of twins in the United States.” Harvard Dataverse. 10.7910/DVN/MAYZE1. Deposited 19 August 2026. PMC13578378 42709798

[46] Z. Griliches, Sibling models and data in economics: Beginnings of a survey. J. Polit. Econ. **87**, S37–S64 (1979).

[47] O. Ashenfelter, A. Krueger, Estimates of the economic return to schooling from a new sample of twins. Am. Econ. Rev. **84**, 1157 (1994).

[48] D. Card, “The causal effect of education on earnings” in Handbook of Labor Economics, O. Ashenfelter, D. Card, Eds. (Elsevier, Amsterdam, The Netherlands, 1999), **vol. 3**, pp. 1801–1863.

[49] A. Björklund, M. Lindahl, E. Plug, The origins of intergenerational associations: Lessons from Swedish adoption data. Q. J. Econ. **121**, 999–1028 (2006).

[50] S. E. Black, P. Devereux, P. Lundborg, K. Majlesi, Poor little rich kids? The role of nature versus nurture in wealth and other economic outcomes and behaviours. Rev. Econ. Stud. **87**, 1683–1725 (2020).

